# Construction of cell factory capable of efficiently converting l-tryptophan into 5-hydroxytryptamine

**DOI:** 10.1186/s12934-022-01745-0

**Published:** 2022-03-24

**Authors:** Yingying Wang, Xueman Chen, Qiaoyu Chen, Ning Zhou, Xin Wang, Alei Zhang, Kequan Chen, Pingkai Ouyang

**Affiliations:** 1 State Key Laboratory of Materials-Oriented Chemical Engineering, Nanjing Tech University, Nanjing, 211816 China; 2 College of Biotechnology and Pharmaceutical Engineering, Nanjing Tech University, Nanjing, 211816 China

## Abstract

**Background:**

l-Tryptophan (l-Trp) derivatives such as 5-hydroxytryptophan (5-HTP) and 5-hydroxytryptamine (5-HT), N-Acetyl-5-hydroxytryptamine and melatonin are important molecules with pharmaceutical interest. Among, 5-HT is an inhibitory neurotransmitter with proven benefits for treating the symptoms of depression. At present, 5-HT depends on plant extraction and chemical synthesis, which limits its mass production and causes environmental problems. Therefore, it is necessary to develop an efficient, green and sustainable biosynthesis method to produce 5-HT.

**Results:**

Here we propose a one-pot production of 5-HT from l-Trp via two enzyme cascades for the first time. First, a chassis cell that can convert l-Trp into 5-HTP was constructed by heterologous expression of tryptophan hydroxylase from *Schistosoma mansoni* (*Sm*TPH) and an artificial endogenous tetrahydrobiopterin (BH_4_) module. Then, dopa decarboxylase from *Harminia axyridis* (*Ha*DDC), which can specifically catalyse 5-HTP to 5-HT, was used for 5-HT production. The cell factory, *E. coli* BL21(DE3)△tnaA/BH_4_/*Ha*DDC-*Sm*TPH, which contains *Sm*TPH and *Ha*DDC, was constructed for 5-HT synthesis. The highest concentration of 5-HT reached 414.5 ± 1.6 mg/L (with conversion rate of 25.9 mol%) at the optimal conditions (substrate concentration,2 g/L; induced temperature, 25℃; IPTG concentration, 0.5 mM; catalysis temperature, 30℃; catalysis time, 72 h).

**Conclusions:**

This protocol provided an efficient one-pot method for converting. l-Trp into 5-HT production, which opens up possibilities for the practical biosynthesis of natural 5-HT at an industrial scale.

**Supplementary Information:**

The online version contains supplementary material available at 10.1186/s12934-022-01745-0.

## Background

l-Tryptophan (l-Trp) is an essential amino acid, and its derivatives, such as l-5-hydroxytryptophan (5-HTP), 5-hydroxytryptamine (5-HT), *N*-acetyl-5-hydroxytryptamine and melatonin, have gained increasing attention due to their direct health and medical benefits, and their production of other valuable molecules as key biosynthetic precursors [1–4]. Among these, 5-HT is a major neurotransmitter that is naturally present in animals and plants [5]. Similar to the catecholamines, dopamine, epinephrine, and norepinephrine, 5-HT modulates the activity of the nervous system and plays a significant role in the coordination of movement and the regulation of mood. As well as scavenging harmful free radicals, 5-HT also plays roles in behaviour management, sleep cycles, appetite and liver regeneration [6].

Currently, the main supply of 5-HT depends on the extraction from mammals (e.g. buffalo, rat, and pig) or plants (safflower, sea buckthorn, and *Moringa Oleifera* seeds) [7]. However, the low 5-HT yield from natural extraction and the insufficient supply of raw materials result in demands that exceed the supply. With the rapid development of biotechnology, biological approaches show great potential for synthesizing natural and non-natural molecules. Previous studies have shown that 5-HT can be produced from l-Trp in the nervous system of human and animals via two enzymatic steps: l-Trp is hydroxylated to 5-HTP by pterin-dependent tryptophan hydroxylase (TPH), then converted to 5-HT by tryptophan decarboxylase (TDC), which is one of the Aromatic amino acid decarboxylase [8]. However, the protocol has several issues, including the problems that TPH possesses poor stability and the expensive cofactor, pterin, is required [5, 9]. Aromatic amino acid decarboxylases (AAADs) play important roles in the key step of 5-HTP conversion to 5-HT. Previously, tryptophan decarboxylase (TDC) was regarded as an enzyme that catalyses non-specific reactions in l-Trp or 5-HTP (prefer to l-Trp), with the result that a one-pot cascade reaction cannot be performed and has therefore never been reported [10, 11] (All the methods are shown in Table 1).

**Table 1:** Different methods to produce 5-HT

Method	Source	Protocol	Yield	References
Natural extraction	Sea buckthorn	Ethanol extraction	4.4 mg/g	[7]
	Rabbit serum	Extraction	3.8 μg/g	[7]
	Adult serum	Extraction	0.1 μg/g	[7]
	Safflower	Ethanol extraction	-	[11]
Biosynthesis	*E.coli*	Fermentation	154.3 mg/L	[6]
	*E.coli*	Whole cell catalysis	24 mg/L	[10]

Dopa decarboxylase (DDC), another AAAD, is able to convert l-dopa to dopamine [12]. In addition, previous study reported that DDC can catalyse 5-HTP into 5-HT without the ability of converting l-Trp [13, 14]. However, almost studies have focused on the characterization of the enzyme involved in the biosynthesis of dopamine, and systematic studies for the production of 5-HT have not been conducted [7, 15].

In this study, we proposed a one-pot enzymatic catalytic approach for converting l-Trp into 5-HT using a whole-cell factory for the first time. First, artificial endogenous cofactor tetrahydrobiopterin (BH_4_) and tetrahydromonapterin (MH_4_) modules were constructed and compared to catalyse l-trp into 5-HTP using a tryptophan hydroxylase (TPH) from *Schistosoma mansoni* with high stability and catalytic ability. Then, a DDC from *Harmonia axyridis* that specifically catalyses 5-HTP to produce 5-HT was introduced to the chassis cell to form the cell factory. The optimal conditions of the recombinant cell for 5-HT production were also studied.

## Results and discussion

### Construction of chassis cells for 5-HTP production

**Fig. 1:**
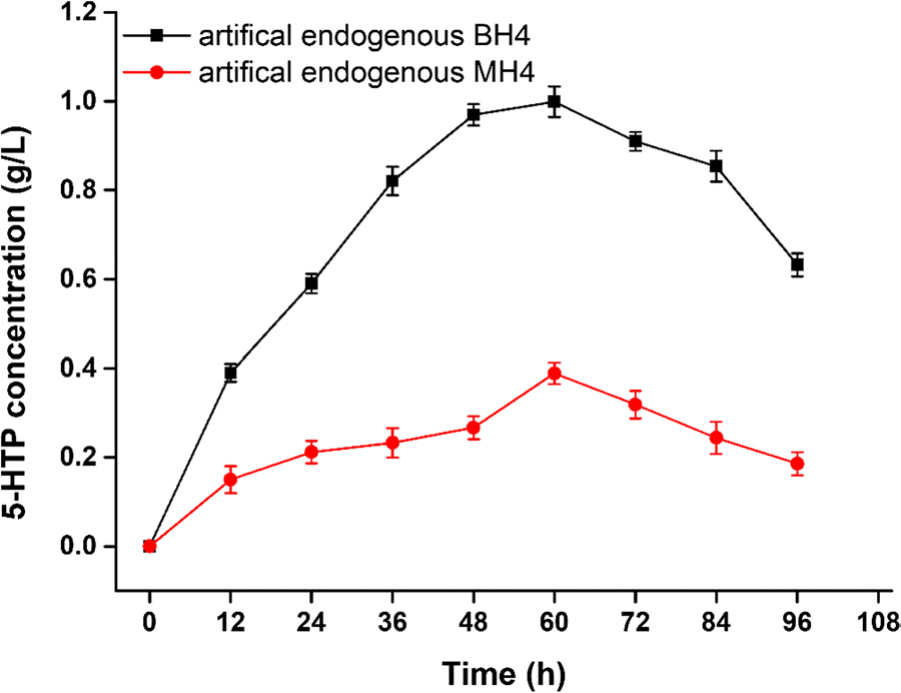
The effect of artificial endogenous cofactors BH_4_ and MH_4_ on 5-HTP synthesis. Experiments were conducted at 37℃, pH 7.0, and 200 rpm for 4 days. The data represent the results of assays performed in triplicate and are presented as the average and standard deviation

To produce 5-HT from l-Trp, a chassis cell that could efficiently convert l-Trp to the intermediate product 5-HTP, needed to be constructed first. Previous studies have reported that TPH from *S. mansoni* (*Sm*TPH) possesses good activity and stability compared that of other resources [16]; therefore, *Sm*TPH was chosen for converting l-Trp into 5-HTP in this study. However, *Sm*TPH is a cofactor-dependent enzyme and requires the expensive cofactor biopterin [9, 17]. To solve this issue, synthesis and regeneration cofactor tetrahydrobiopterin (BH_4_) and tetrahydromonapterin (MH_4_) modules were constructed in the chassis cell containing *Sm*TPH [1, 18]. As shown in Fig. 1, the 5-HTP concentrations in the cells containing BH_4_ and MH_4_ increased until 60 h of fermentation, then decreased with the further increase of time. The observed decrease in 5-HTP concentration may have been due to the oxidation and degradation of 5-HTP as the fermentation time increased [5]. The highest concentrations of 5-HTP were 0.93 g/L and 0.34 g/L at 60 h with BH_4_ and MH_4_, respectively. Zhang et al. reported that the production of 5-HTP from *Sm*TPH using BH_4_ as a cofactor was higher than that when using MH_4_ as a cofactor in *S. cerevisiae*, which correlated with the findings of our study [18]. Therefore, the chassis cell containing the synthesis and regeneration cofactor BH_4_ and *Sm*TPH was used in the next experiment.

### Expression and purification of dopa decarboxylase HaDDC and its catalytic specificity towards L-Trp and 5-HTP

Dopa decarboxylase from Drosophila melanogaster (*dr*DDC) has attracted significant attention, due to its activity for both 5-HTP and l-DOPA but with no activity to l-Trp [19–21]. In this study, we used the DDC from *Harmonia axyridis* (*Ha*DDC, GenBank: AMQ 13055.1) for 5-HT synthesis.

As shown in Additional file 1: Fig. S2, SDS-PAGE analysis revealed that the recombinant *Ha*DDC was approximately 55 kD, indicating that *Ha*DDC was successfully expressed as an active form in *E. coli* BL21(DE3)△tnaA. The recombinant *Ha*DDC was purified by a Ni–NTA resin, with a recovery yield of 80.3%.

**Fig. 2:**
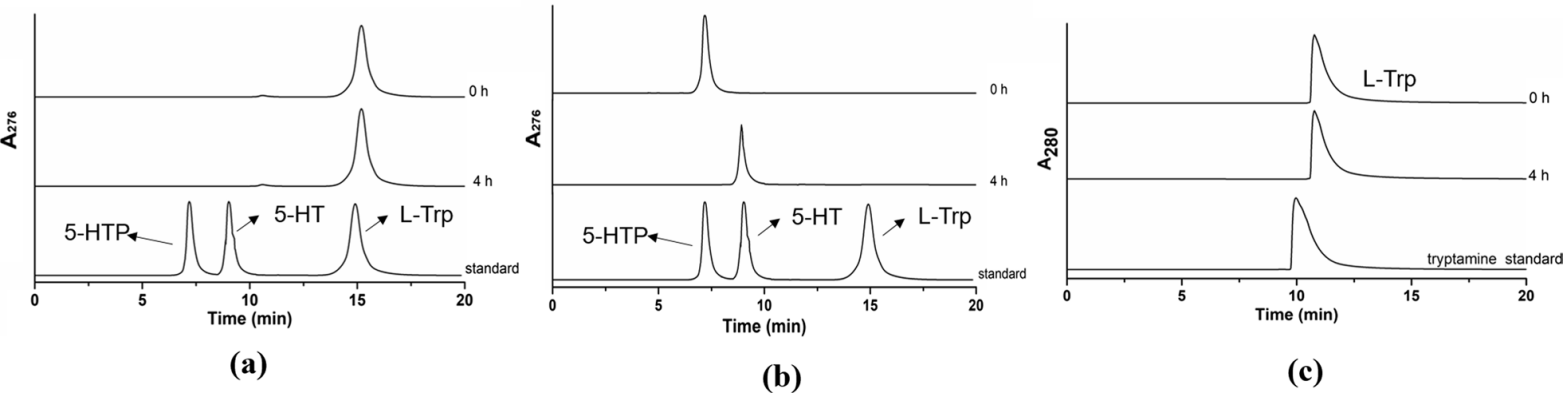
HPLC analysis of *Ha*DDC catalytic product toward L-Trp (a) and 5-HTP (b), tryptamine standard (c)

To evaluate the catalytic specificity of purified *Ha*DDC, l-Trp and 5-HTP were used as substrates respectively. As shown in Fig. 2a, no 5-HT was released from l-Trp within 4 h or longer (data not shown), indicating that the recombinant *Ha*DDC had no activity toward l-Trp. Meanwhile, as shown in the Fig. 2c, there was also no tryptamine found from l-Trp. It was clearly that 5-HT was released within 4 h by *Ha*DDC with 5-HTP as a substrate in the Fig. 2b. These results showed that *Ha*DDC exhibited strict substrate specificity similar to that displayed by *dr*DDC from *D. melanogaster* [7, 15]. According to the results shown in Table 2, *Ha*DDC exhibited a high efficiency on 5-HTP with a yield of 88.45% at 4 h, which was much higher than that of TDC (16.04%) from Catharanthus roseus which was higher than TDC reported previously [6, 11]. Therefore, *Ha*DDC from *H. axyridis* was chosen for converting 5-HTP into 5-HT (Additional file 1: Figs. S1–S7, Table S1).

**Table 2:** Substrate specificity of *Ha*DDC

Substrate	Concentration (mM)	Time (h)	Conversion yield (mol%)
L-Trp	200 ± 0.13	4	0
5-HTP	200 ± 0.14	4	88.45

### One-pot catalysis for 5-HT production

**Fig. 3:**
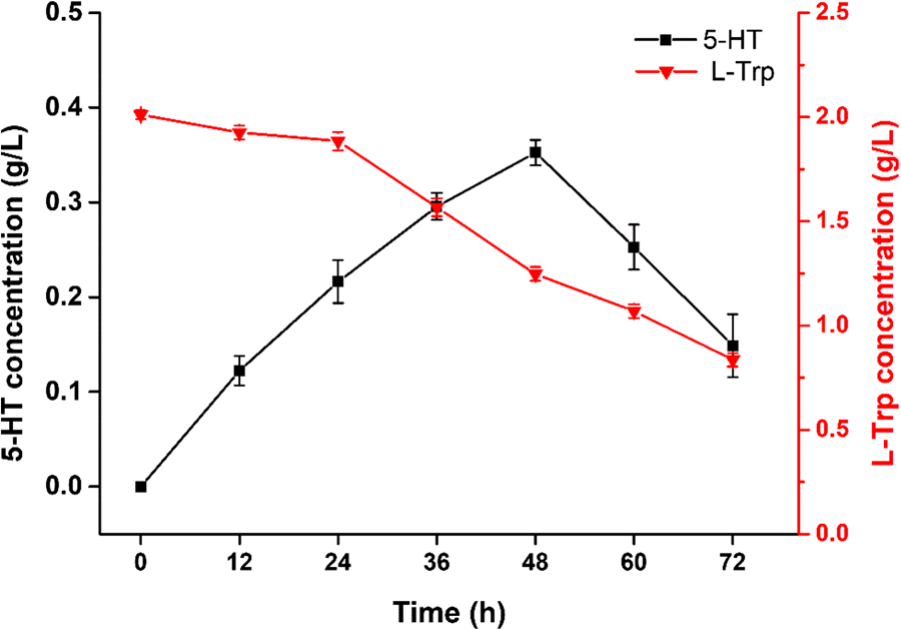
Time course of 5-HT production from recombinant *Sm*TPH and *Ha*DDC in vitro. Experiments were conducted with purified recombinant *Sm*TPH and *Ha*DDC in vitro at 37℃, pH 7.2, and 200 rpm for 72 h. Sampling and measuring were conducted every 12 h. Data represent the results of assays performed in triplicate and are presented as the average and standard deviation

**Fig. 4:**
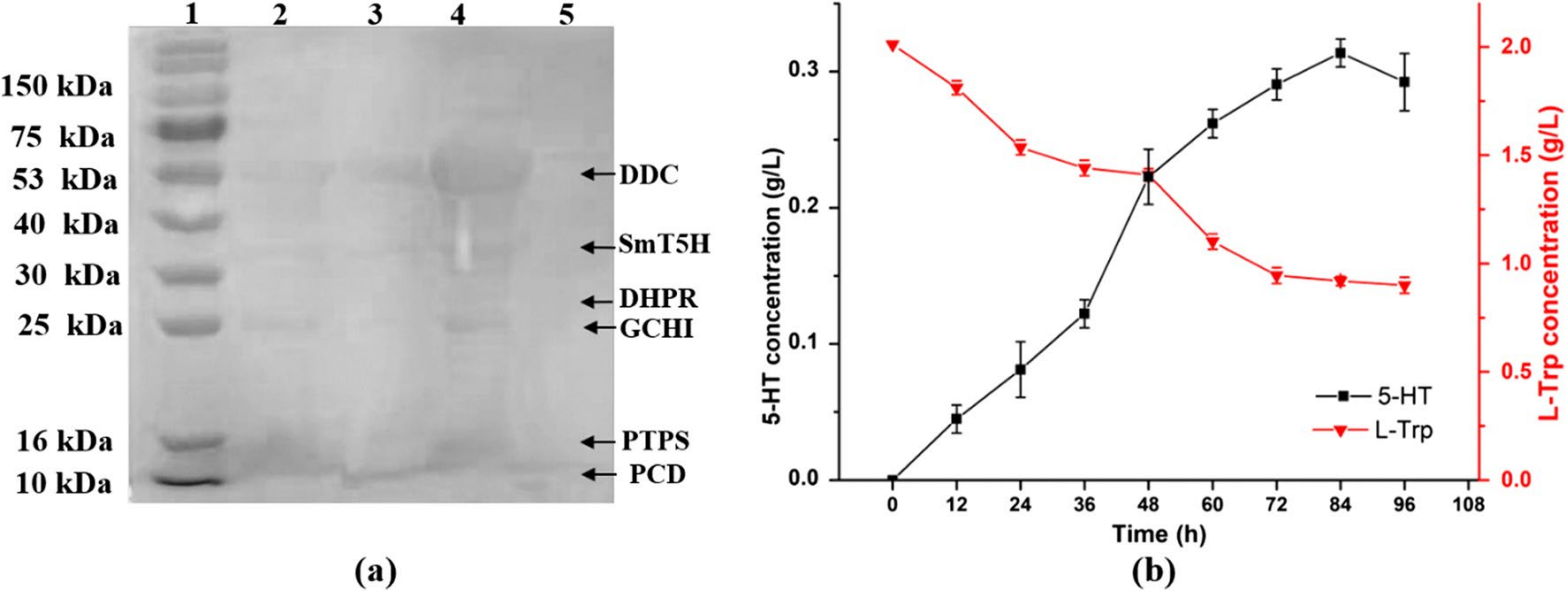
Enzyme cascade of *Sm*TPH and *Ha*DDC to produce 5-HT in vivo. (a) Protein expression in *E. coli* BL21(DE3)△tnaA/*Sm*TPH-*Ha*DDC/BH_4_ and *E. coli* BL21(DE3)△tnaA/*Ha*DDC-*Sm*TPH/BH_4_. Lane 1. marker; Lanes 2 & 3. soluble and inclusion expression of *E. coli* BL21(DE3)△tnaA/*Sm*TPH-*Ha*DDC/BH_4_; Lanes 4 & 5, soluble and inclusion expression of *E. coli* BL21(DE3)△tnaA/*Ha*DDC-*Sm*TPH/BH_4_. (b) Batch production of 5-HT using *E. coli* BL21(DE3)△tnaA/*Ha*DDC-*Sm*TPH/BH_4_

The enzyme cascade of purified *Sm*TPH and *Ha*DDC was investigated both in vitro and in vivo. The catalysis processing of l-Trp by recombinant *Sm*TPH and *Ha*DDC in vitro showed that 5-HT production increased along with increasing time before 48 h, then decreased with the further increase of time (Fig. 3). Meanwhile, the concentration of l-Trp decreased continuously. The highest concentration of 5-HT was 352.4 mg/L, with a yield of 17.62%, at 48 h, which was similar to the two steps to produce 5-HT [6]. The in vivo studies of *Sm*TPH and *Ha*DDC were investigated with *Escherichia coli* BL21(DE3)△tnaA/BH_4_/*Ha*DDC-*Sm*TPH and *E. coli* BL21(DE3)△tnaA/BH_4_/*Sm*TPH-*Ha*DDC, respectively. The production of 5-HT was obtained in the former but not in the latter (data not shown). To explore the reasons, SDS-PAGE was used to analyse the protein expression of *E. coli* BL21(DE3)△tnaA/BH_4_/*Ha*DDC-*Sm*TPH and *E. coli* BL21(DE3)△tnaA/BH_4_/*Sm*TPH-*Ha*DDC. As shown in Fig. 4a, *E. coli* BL21(DE3)△tnaA/BH_4_/*Ha*DDC-*Sm*TPH exhibited better protein expression than *E. coli* BL21(DE3)△tnaA/BH_4_/*Sm*TPH-*Ha*DDC with *Ha*DDC in the second multiple cloning site. This phenomenon may be attributed to the insert position of the *Ha*DDC gene, which affects the expression of both enzymes. Thus, *E. coli* BL21(DE3)△tnaA/BH_4_/*Ha*DDC-*Sm*TPH was chosen to produce 5-HT from l-Trp. As shown in Fig. 4b, the l-Trp concentration decreased continuously from 2.00 to 0.899 g/L within 96 h. Meanwhile, the 5-HT concentration increased as time increased and reached the highest concentration (0.3136 g/L) at 84 h, which was 2.04-fold higher than that obtained from the two-stage fermentation process by *Ct*AAAH and TDC [6]. However, the concentration of 5-HT decreased after 84 h and the fermentation broth turned dark, which was similar to the results reported previously [22]. It may because 5-HT was unstable. During the reaction process, oxidation reaction occurred and degradation products generated, resulting in the decrease of 5-HT and colour changed. In addition, l-Trp could be also degraded by other tryptophanases in *E.coli* and caused the broth turned dark [22–24].

### *Optimisation of one-pot cascade *in vivo

**Fig. 5:**
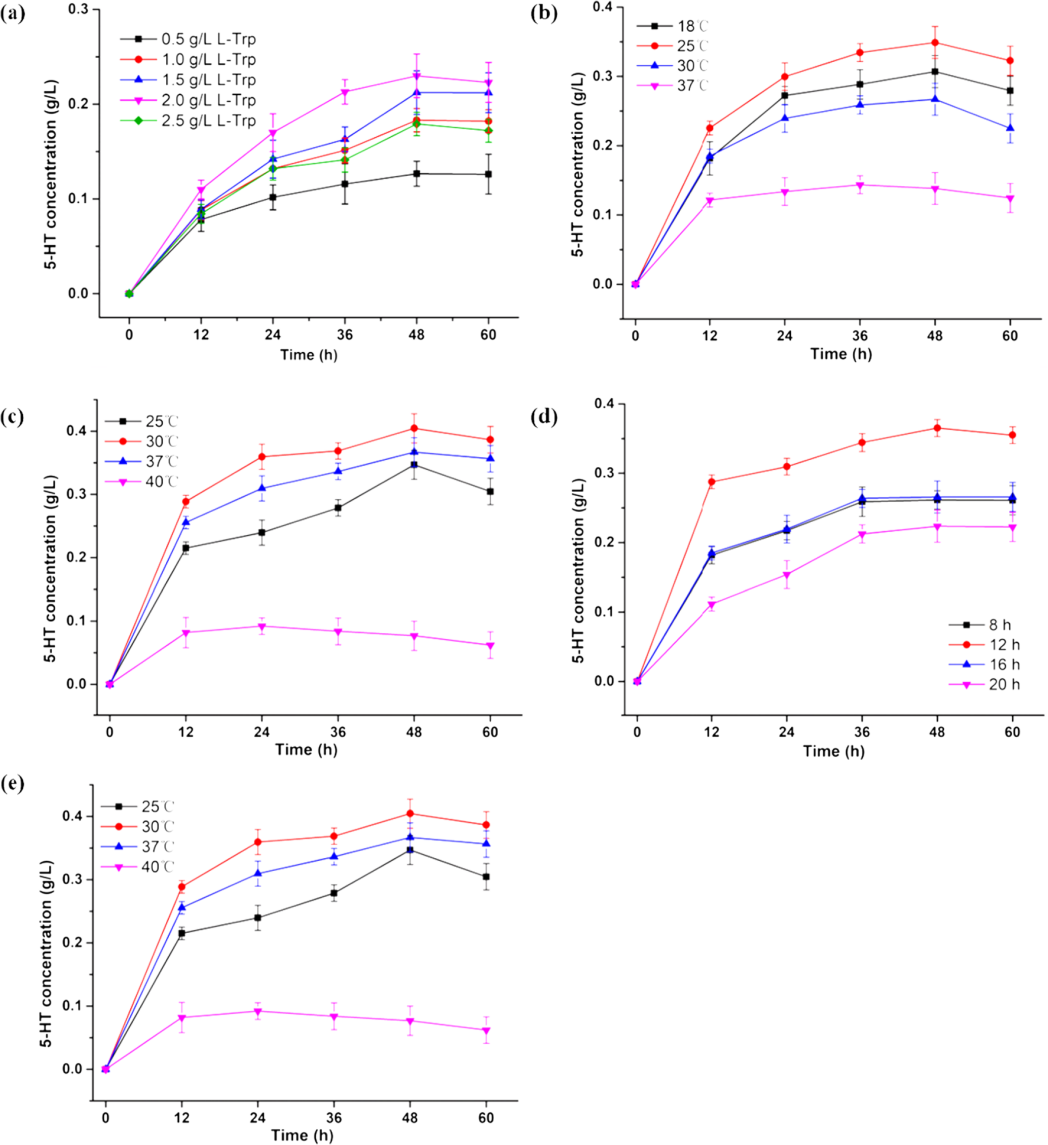
The effects of induction temperature, induction time, IPTG concentration and catalysis temperature on 5-HT synthesis. **a** Optimal L-Trp concentration for 5-HT synthesis. **b** Optimal culture temperature for 5-HT synthesis. **c** Optimal IPTG concentration for 5-HT synthesis. **d** Optimal induction time for 5-HT synthesis. **e** Optimal catalysis temperature for 5-HT synthesis. Aliquots of solution were taken and diluted for HPLC analysis every 12 h

To determine the optimal conditions for 5-HT production, a number of catalysis conditions were investigated. The effect of l-Trp concentration on 5-HT production was illustrated in Fig. 5a. 5-HT concentration increased with l-Trp concentration from 0.5 to 2.0 g/L before 48 h, followed by decreasing slightly. However, as the concentration of l-Trp increased to 2.5 g/L, the 5-HT concentration lower than that from 1.0, 1.5, 2.0 g/L l-Trp. Therefore, 2.0 g/L l-Trp was chosen for subsequent experiments.

The effect of induction temperature on the production of 5-HT is shown in Fig. 5b. The production of 5-HT increased quickly as the induction temperature increased from 18 to 25 ℃, then decreased with the temperature increased from 25 to 37 ℃. Therefore, the optimal induction temperature for the production of 5-HT was 25 ℃.

The effects of concentration of IPTG on 5-HT synthesis were investigated (Fig. 5c). 5-HT concentration increased with the increase in concentration of IPTG from 0.025 to 0.05 mM, followed by a decrease at higher IPTG concentrations. The results suggested that 0.05 mM was the optimal IPTG concentration.

As shown in Fig. 5d, an increase in the concentration of 5-HT in the broth appeared at 8 h to 12 h during incubation with *E. coli* BL21(DE3)△tnaA/BH_4_/*Ha*DDC-*Sm*TPH, then decreased. Thus, the optimal time for induction was 12 h.

The relationship between 5-HT concentration and catalysis temperature was presented in Fig. 5e. 5-HT production from l-Trp increased as the catalysis temperature increased from 25 to 30 ℃, and the maximum concentration of 5-HT (0.4145 ± 1.6 g/L, reduced L-Trp was 1.13 g/L) was reached at 30℃. However, with the temperature increased from 30 to 40℃, the yield of the product decreased rapidly.

Based on these results, the optimal conditions for 5-HT production of one-pot catalysis using *E. coli* BL21(DE3)△tnaA/BH_4_/*Ha*DDC-*Sm*TPH were 12 h for the expression of proteins at 25 ℃ with 0.05 mM IPTG, and the optimal conditions for catalysis were 30 ℃ for 48 h. The highest yield of 5-HT from l-trp was 25.9 mol%.

## Conclusions

In this study, a cell factory capable of efficiently converting l-Trp into 5-HT was successfully constructed. First, a chassis cell that contained a screened artificial endogenous BH4 module as a cofactor and tryptophan hydroxylase from *Schistosoma mansoni* (*Sm*TPH) was constructed, which could convert L-Trp into 5-HTP efficiently. The dopa decarboxylase from *Harminia axyridis* (*Ha*DDC) that can specific catalyse 5-HTP to 5-HT was chosen for 5-HT production. Finally, the two enzymes were cascaded in vivo for converting L-Trp to 5-HT, which lead to a yield of 414.5 ± 1.6 mg/L at the optimal conditions. This study provides a novel and important foundation for artificial cell factory synthesis of 5-HT.

## Methods

### Chemicals

l-Tryptophan (l-Trp), 5-hydroxytryptophan (5-HTP) and 5-hydroxytryptamine (5-HT) standards were purchased from Aladdin Industrial Corporation (Shanghai, China). (6R)-l-erythro-5, 6, 7, 8-tetrahydrobiopterin (BH_4_), pyridoxal 5-phosphatemonohydrate (PLP) were purchased from Shanghai Yuanye Bio-Technology Co., Ltd. (Shanghai, China). Other chemicals and solvents in this study were purchased from local suppliers and were of analytical grade or higher purity.

### Plasmid and strain construction

*E.coli* Trans1T1 was used for plasmid construction and propagation, and *E. coli* BL21(DE3) was used for protein expression. As tryptophanase A (tnaA) gene, which encodes tryptophanase, is necessary to accelerate degrade l-Trp and 5-HTP into indole and 5-hydroxy indole in *E.coli*, respectively. Therefore, tnaA gene should be knocked out in *E. coli* BL21(DE3) to ensure the biosynthesis of 5-HTP or 5-HT, using the previously established CRISPR/Cas9 protocol [1, 25]. The expression vectors pCDFDuet-1 and pET-28a ( +) were supplied by Novagen Co., Ltd.

Plasmid construction and DNA manipulation were performed following the standard molecular cloning protocols. First, the PTPS, SPR, PCD, DHPR human genes and mtrA from *Bacillus subtilus* were codon-optimized, synthesized and inserted into pCDF-Duet-1 to acquire *E. coli* BL21(DE3)△tnaA/BH_4_ [1]. Another cofactor, tetrahydromonapterin (MH_4_), whose relative genes were also constructed in pCDF-Duet-1, was used to acquire *E. coli* BL21(DE3)△tnaA/MH4 according to a previous study [18]. *Sm*TPH from *Schistosoma mansoni* was codon-optimised for *E. coli* expression, synthesised, and inserted into pET-28a( +), then transferred into *E. coli* BL21(DE3)△tnaA/BH_4_ and *E. coli* BL21(DE3)△tnaA/MH_4_ to acquire *E. coli* BL21(DE3)△tnaA/BH_4_/*Sm*TPH and *E. coli* BL21(DE3)△tnaA/MH_4_/*Sm*TPH. *Ha*DDC from *Harminia axyridis* was also codon- optimised for *E. coli* expression, synthesized and inserted into pET-28a ( +), then transferred into *E. coli* BL21(DE3)△tnaA to acquire *E. coli* BL21(DE3)△tnaA/*Ha*DDC.

The gene fragment of *Ha*DDC was amplified from pET-28a ( +)-*Ha*DDC using the primer pairs, *Ha*DDC-F-BamHI, *Ha*DDC-R-HindIII, *Ha*DDC-F-EcoRI, and *Ha*DDC-R-SalI. First, BamHI- and HindIII-digested polymerase chain reaction (PCR) products of *Ha*DDC were ligated to BamHI- and HindIII- digested pET28a ( +)-*Sm*TPH and EcoRI- and SalI- digested pET28a ( +)-*Sm*TPH to construct the plasmids pET28a ( +)-*Sm*TPH-*Ha*DDC and pET28a ( +)-*Ha*DDC-SmTPH. The plasmids were constructed in *E. coli* Trans1T1 and transferred into *E. coli* BL21(DE3)△tnaA/BH_4_ a to acquire *E. coli* BL21(DE3)△tnaA/BH_4_/*Sm*TPH/*Ha*DDC and *E. coli* BL21(DE3)△tnaA /BH_4_/*Ha*DDC/*Sm*TPH. The plasmids and strains along with the primers and specific structures of the plasmids, are shown in Table 3.

**Table 3:** List of strains and plasmids used for the production of 5-HTP and 5-HT

Name	Description	References
Strains		
*E. coli* BL21(DE3)	Protein expression host	This lab
*E. coli* BL21 (DE3)△tnaA	*E. coli* BL21(DE3)△tnaA	This study
*E. coli* Trans 1T1	Cloning host	This study
*E. coli* BL21(DE3) △tnaA /BH_4_/*Sm*TPH	*E. coli* BL21(DE3)△tnaA/pCDFDuet-BH_4_/pET-28a( +)-*Sm*TPH	This study
*E. coli* BL21(DE3) △tnaA/MH_4_/*Sm*TPH	*E. coli* BL21(DE3)△tnaA/pCDFDuet-MH_4_/pET-28a( +)-*Sm*TPH	This study
*E. coli* BL21(DE3) △tnaA/*Ha*DDC	*E. coli* BL21(DE3)△tnaA/pET-28a( +)-*Sm*TPH	This study
*E. coli* BL21(DE3) △tnaA/BH_4_/*Sm*TPH/*Ha*DDC	*E. coli* BL21(DE3)△tnaA/pCDFDuet-BH_4_/pET-28a( +)-*Sm*TPH-*Ha*DDC	This study
*E. coli* BL21(DE3) △tnaA/BH_4_/*Ha*DDC/*Sm*TPH	*E. coli* BL21(DE3)△tnaA/pCDFDuet-BH_4_/pET-28a( +)- *Ha*DDC-*Sm*TPH	This study
Plasmids		
pET-28a ( +)	ColE1 ori, Kan^R^, *E. coli* expression vector	This lab
pET-28a ( +)-*Sm*TPH	pET-28a ( +); tryptophan hydroxylase gene from *S.mansoni*	This study
pCDFDuet-1	CloDF13 ori, Sm^R^, *E. coli* expression vector	This lab
pCDFDuet-BH_4_	pCDFDuet-1; pterin-4-alpha-carbinolamine dehydratase (PCD), dihydropteridine reductase (DHPR), 6-pyruvate-tetrahydropterin synthase (PTPS), sepiapterin reductase (SPR), GTP cyclohydrolase I (GCHI)	[1]
pCDFDuet-MH_4_	pterin-4-alpha-carbinolamine dehydratase (PCD) and dihydropteridine reductase (DHPR) from human	[18]
pET-28a ( +)-*HaDDC*	pET-28a ( +); dopa decarboxylase from *H.axyridis*	This study
pET-28a ( +)-*Ha*DDC-*Sm*TPH	pET-28a ( +); dopa decarboxylase from *H. axyridis*; tryptophan hydroxylase gene from *S. mansoni*	This study
pET-28a ( +)-*Sm*TPH- *Ha*DDC	pET-28a ( +); tryptophan hydroxylase gene from *S. mansoni;* dopa decarboxylase from *H. axyridis*	This study
Primers		
*Ha*DDC-F-BamHI	GGATCCGAATTCATGGAGGCG	This study
*Ha*DDC -R-HindIII	AAGCTTTTATTCACCCAGGATATC	This study
*Ha*DDC-F-EcoRI	GAATTCATGGAGGCGAACCAG	This study
*Ha*DDC-R-SalI	GTCGACTTATTCACCCAGGATATCGTCC	This study

Luria–Bertani (LB) medium was used for cell cultivation and enzyme expression [26]. Modified M9 medium (M9Y) was used for in vivo hydroxylation of l-Trp to 5-HTP [27]. All the single colonies of the recombinant strains were separately inoculated into 5 mL of LB media containing 25 mg/L kanamycin and/or streptomycin and incubated at 37℃ and mixed at 200 rpm for 12 h. Cultures (1 mL) were then inoculated into 100 mL of LB media at 37℃. When the OD_600_ reached 0.4–0.6, the cells were induced with isopropyl-β-d-thiogalactoside (IPTG) for 12 h at either 25℃ (*E. coli* BL21(DE3)△tnaA/BH_4_/SmTPH) or 18℃ (*E. coli* BL21(DE3)△tnaA/*Ha*DDC).

### The effect of cofactor BH_4_ and MH_4_ synthesis and regeneration pathways on the conversion of L-Trp into 5-HTP

The fermentation broth of *E. coli* BL21(DE3)△tnaA/BH_4_/*Sm*TPH and *E. coli* BL21(DE3)△tnaA/MH_4_/*Sm*TPH were centrifuged at 4000 rpm for 10 min, and the cells were respectively transferred to M9Y media containing 2 g/L of l-Trp*.* Sampling and measuring were conducted at 12-h intervals.

## Purification of E. coli BL21(DE3)△tnaA/HaDDC and its catalysis specificity towards L-Trp and 5-HTP

The cells harvested from cultures were washed, resuspended in 50 mM HEPES (pH 7.0), and lysed at 4 ℃ by JY92-IIN ultrasonication (Ningbo Xinzhi Biotechnology, Ltd., Ningbo, China). The lysates were centrifuged at 12,000 rpm at 4 ℃ for 20 min, and the supernatant was used as a crude enzyme solution. The recombinant *Ha*DDC was purified using a fast protein liquid chromatography (FPLC) system (GE AKTA Pure 150; General Electric Co., Fairfield, America) with a Ni-nitrilotriacetic acid affinity chromatography (Ni–NTA) column (His Trap™ FF5 mL) according to the manufacturer’s instructions. Protein concentrations were determined at 595 nm using the Bradford method with bovine serum albumin as the standard [28]. All protein samples were analysed by reductive SDS-PAGE with 20 mM β-Mercapto ethanol incubation. A premixed protein marker (Takara Biotechnology Co., Ltd., Nanjing, China) containing 180-, 140-, 100-, 75-, 60- and 45-kDa proteins was used as the molecular mass standard. The method of purification for the recombinant protein *Sm*TPH was same as that for *Ha*DDC.

The recombinant *Ha*DDC activity was assayed at 35 ℃ in a reaction mixture of 2 mL, containing 50 mM HEPES (pH 7.0), 0.4 mM PLP, 50 μL purified *Ha*DDC (protein concentration of 15 g/L) and 200 μM L-Trp or 5-HTP.

### One-pot catalysis for 5-HT production in vitro and in vivo

The in vitro cascade of *Sm*TPH and *Ha*DDC was conducted in a reaction system (2 mL) that contained 7 mM DTT, 0.1 M ferrous ammonium sulfate, 0.1 mg/mL catalase, 0.6 mM BH_4_, 0.1 mM PLP, 0.5 mM L-Trp, 100 µL purified *Sm*TPH (1.0 g/L), 100 µL purified *Ha*DDC (1.0 g/L) and 50 mM HEPES (pH 7.0), and incubated at 30 ℃ [1, 7]. In addition to these cofactors, reactions were supplemented with catalase and dithiothreitol to protect against oxidative degradation [18].

The in vivo cascade of *Sm*TPH and *Ha*DDC was performed as follows: the *E. coli* BL21(DE3)△tnaA/BH_4_/*Sm*TPH/*Ha*DDC and *E. coli* BL21(DE3)△tnaA /BH_4_/*Ha*DDC/*Sm*TPH fermentation broths were centrifuged at 4000 rpm for 10 min and the cells (final cell concentration of 3.8 OD/mL) were transferred to 100 mL of M9Y media containing 2 g/L L-Trp [1]. Cells were incubated at pH 7.0 and 30 ℃. Sampling and measuring were conducted every 12 h.

### Optimisation of the production of 5-HT from L-Trp using *E.coli* BL21(DE3)△tnaA /BH_4_/*Ha*DDC-*Sm*TPH whole-cell factory

Cells were transferred to 100 mL of M9Y medium in a 500 mL flask and shaken at 200 rpm in various conditions.

In this study, single-factor experiments using five predominant factors (substrate concentration, IPTG, induced time, induced temperature and catalysis temperature) were conducted to investigate the effects of these factors on the production of 5-HT [29]. Investigation of the effect of each factor on the production of 5-HT was carried out based on changes only to that factor, while keeping other variables constant.

The investigation was carried out at varying concentrations of L-Trp (0.5, 1.0,1.5, 2.0 g/L), IPTG (0.25, 0.5, 0.75, and 1 mM), induced time (8 h, 12 h, 16 h and 20 h), induced temperature (18 ℃, 25 ℃, 30 ℃ and 37 ℃), and catalysis temperature (25 ℃, 30 ℃, 37 ℃ and 40 ℃).

### Analytical method

The cell growth density at 600 nm (OD_600_) was measured using an UV1000D ultraviolet–visible spectrophotometer (AOE Instruments, Shanghai Co., Ltd). High-performance liquid chromatographic (HPLC) analysis of the L-Trp, 5-HTP and 5-HT contents was performed on an Agilent 1260 series LC system (Agilent Technologies, Santa Clara, CA, USA) with an ultraviolet detector reading at 276 nm [1]. The samples were filtered through Millex-LG filter units (Millipore, Billerica, MA, USA) prior to HPLC analysis. Separation of samples was achieved using a reverse phase Agilent TC-C18 column (5 × 4.6 mm × 250 mm; Agilent) with a constant flow rate of 1 mL/min at 25 ℃ and an injection volume of 10 μL. The mobile phase consisted of methanol (and potassium phosphate buffer (10 mM, pH 6.5). The substrate and product concentration were measured by HPLC against the l-Trp, 5-HTP and 5-HT standards using a calibration curve. Tryptamine standard was eluted via column chromatography (TC18, 5 × 4.6 mm × 250 mm; Agilent) in the following gradients: methanol (solvent A) and 0.1% formic acid in water (solvent B): 0–15 min 10% A; 15–16 min, 55% A; 16–22 min, 100% A; 22–26 min, 10% A; the flow rate was 0.8 mL/min, and the reaction absorption was recorded at 280 nm.

The 5-HT yield from l-Trp was calculated according to the following equation:

5-HT yield (mol%) = 5-HTP released (mol)/ l-Trp added (mol).

## Supplementary Information

**Additional file 1: Fig. S1.** SDS-PAGE analysis of different induction temperatures on the protein expression of *Sm*TPH. **Fig. S2.** SDS-PAGE of cell extracts of *E.coli* BL21(DE3)/pET28a-DDC. **Fig. S3.** Effects of different induction temperatures on the catalysis in 5-HTP of *Ha*DDC. **Fig. S4.** Effects of different pH on the catalysis in 5-HTP of *Ha*DDC. **Fig. S5.** Effects of different PLP concentration on the catalysis in 5-HTP of *Ha*DDC. **Fig. S6.** Phylogenetic analysis of *Ha*DDC with other DDCs. **Fig. S7.** Plasmids construction of *Sm*TPH gene and *Ha*DDC gene at different cloning sites in the pET-28a ( +). **Table S1.** The synthesized protein sequence applied in this study.

## Data Availability

All data generated or analyzed during this study are included in this article and its Additional file 1.

## Abbreviations

l-Trp: l-Tryptophan
5-HTP: l-5-hydroxytryptophan
5-HT: 5-Hydroxytryptamine
AAAD: Aromatic amino acid decarboxylases
FPLC: Fast protein liquid chromatography
HPLC: High-performance liquid chromatographic
LB: Luria–Bertani
PCR: Polymerase chain reaction
PLP: Pyridoxal5-phosphatemonohydrate
