# Correction to: Construction of cell factory capable of efficiently converting l‑tryptophan into 5‑hydroxytryptamine

**DOI:** 10.1186/s12934-023-02102-5

**Published:** 2023-05-02

**Authors:** Yingying Wang, Xueman Chen, Qiaoyu Chen, Ning Zhou, Xin Wang, Alei Zhang, Kequan Chen, Pingkai Ouyang

**Affiliations:** 1 State Key Laboratory of Materials-Oriented Chemical Engineering, Nanjing Tech University, Nanjing, 211816 China; 2 College of Biotechnology and Pharmaceutical Engineering, Nanjing Tech University, Nanjing, 211816 China

**Correction to: Microbial Cell Factories (2022) 21:47**

10.1186/s12934-022-01745-0

Unfortunately, the original publication of the article [1] contained the below errors.

In Fig. 5, the part Fig. 5c was incorrect and found to be the duplication of Fig. 5e. The corrected Fig. 5 is given below.

**Fig. 5:**
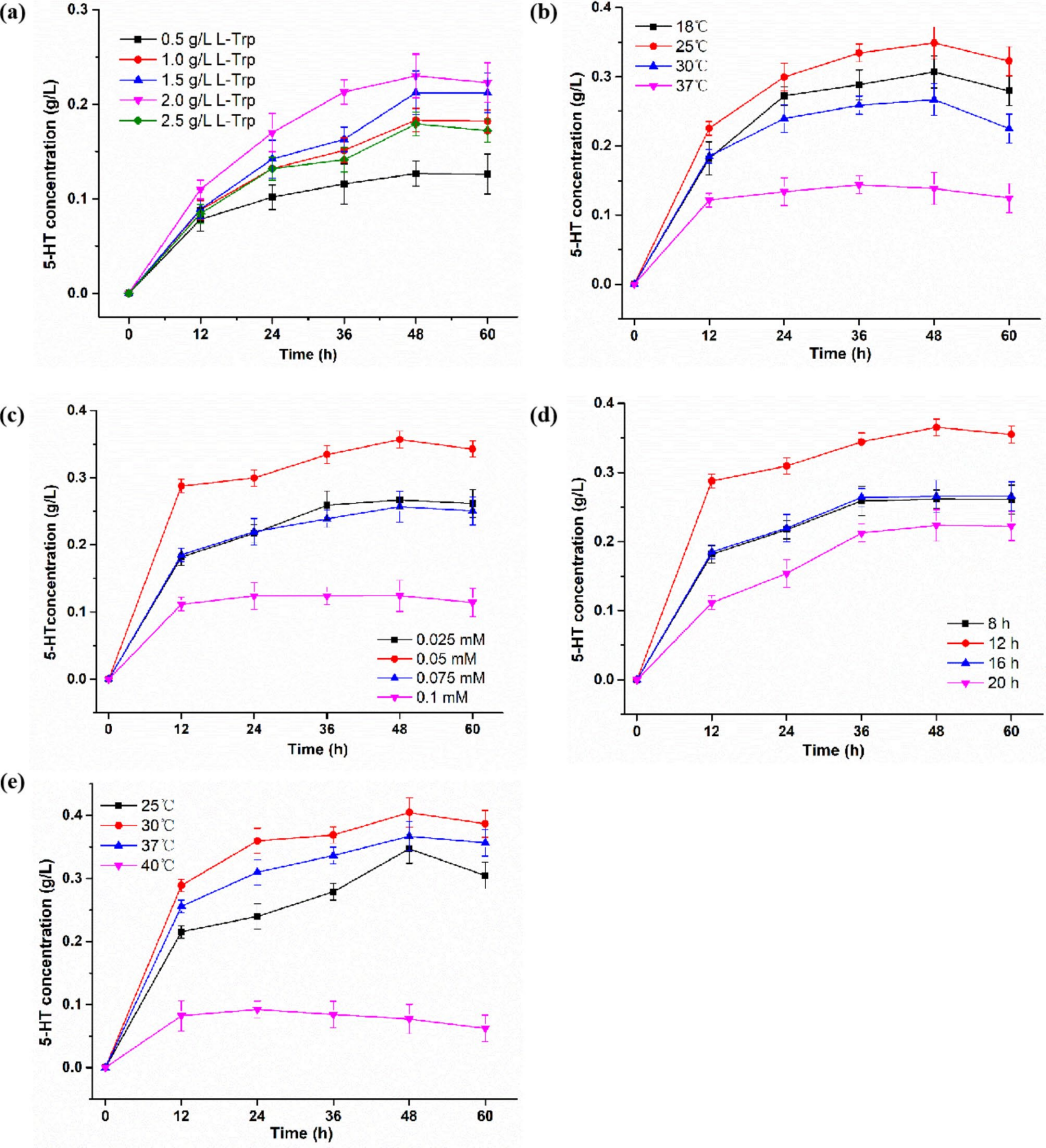
The effects of induction temperature, induction time, IPTG concentration and catalysis temperature on 5-HT synthesis. (a) Optimal L-Trp concentration for 5-HT synthesis. (b) Optimal culture temperature for 5-HT synthesis. (c) Optimal IPTG concentration for 5-HT synthesis. (d) Optimal induction time for 5-HT synthesis. (e) Optimal catalysis temperature for 5-HT synthesis. Aliquots of solution were taken and diluted for HPLC analysis every 12 h

In “Abstract” under the Results section, the IPTG concentration that reads as “concentration, 0.5 mM” should read as “concentration, 0.05 mM”.

In “Methods, under the sub heading “Optimization of the production of 5-HT from L-Trp using *E.coli* BL21 (DE3) △ tnaA/BH4/*Ha*DDC-*Sm*TPH whole cell factory”, the sentence that reads as “The investment was carried out at varying concentration trajectories of L-Trp (0.5, 1.0, 1.5, 2.0 g/L), IPTG (0.25, 0.5, 0.75, and 1 mM)……”, should read as “The investment was carried out at varying concentration trajectories of L-Trp (0.5, 1.0, 1.5, 2.0 g/L), IPTG (0.025, 0.05, 0.075, and 0.1 mM)…….”.

The authors apologize for the mistakes.
