# Supplementary material for: Construction of cell factory capable of efficiently converting l-tryptophan into 5-hydroxytryptamine
Source: Microb Cell Fact. 2022 Mar 24;21:47. doi: 10.1186/s12934-022-01745-0 (PMC8944007; doi:10.1186/s12934-022-01745-0)
Supplement: Supplementary file 1 — Additional file 1: Fig. S1. SDS-PAGE analysis of different induction temperatures on the protein expression of SmTPH. Fig. S2. SDS-PAGE of cell extracts of E.coli BL21(DE3)/pET28a-DDC. Fig. S3. Effects of different induction temperatures on the catalysis in 5-HTP of HaDDC. Fig. S4. Effects of different pH on the catalysis in 5-HTP of HaDDC. Fig. S5. Effects of different PLP concentration on the catalysis in 5-HTP of HaDDC. Fig. S6. Phylogenetic analysis of HaDDC with other DDCs. Fig. S7. Plasmids construction of SmTPH gene and HaDDC gene at different cloning sites in the pET-28a ( +). Table S1. The synthesized protein sequence applied in this study. [file 12934_2022_1745_MOESM1_ESM.docx]

**Supplementary information**

**Construction of cell factory capable of efficiently converting L-tryptophan into 5-hydroxytryptamine**

Yingying Wang, Xueman Chen, Qiaoyu Chen, Ning Zhou, Xin Wang, Alei Zhang, Kequan Chen*, Pingkai Ouyang

State Key Laboratory of Materials-Oriented Chemical Engineering, College of Biotechnology and Pharmaceutical Engineering, Nanjing Tech University, Nanjing 211816, China.

Address correspondence to Kequan Chen, kqchen@njtech.edu.cn

**Fig.S1** SDS-PAGE analysis of different induction temperatures on the protein expression of *Sm*TPH.

**Fig.S2** SDS-PAGE of cell extracts of *E.coli* BL21(DE3)/pET28a-DDC.

**Fig.S3** Effects of different induction temperatures on the catalysis in 5-HTP of *Ha*DDC.

**Fig.S4** Effects of different pH on the catalysis in 5-HTP of *Ha*DDC.

**Fig.S5** Effects of different PLP concentration on the catalysis in 5-HTP of *Ha*DDC.

**Fig.S6** Phylogenetic analysis of *Ha*DDC with other DDCs.

**Fig.S7** Plasmids construction of *Sm*TPH gene and *Ha*DDC gene at different cloning sites in the pET-28a (+).

**Tab. S1** The synthesized protein sequence applied in this study.

***
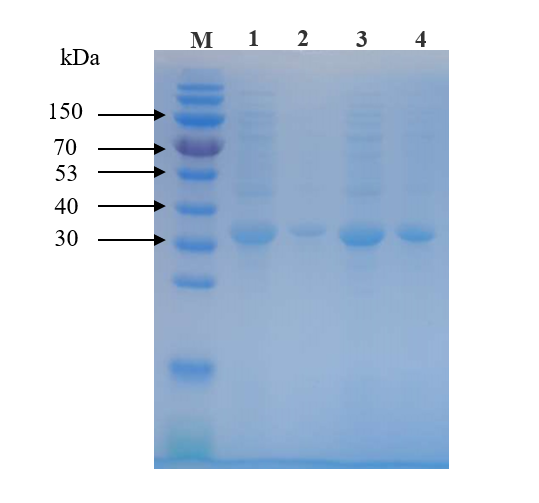
***

Fig. S1. SDS-PAGE analysis of different induction temperatures on the protein expression of *Sm*TPH. M, Marker; Lanes 1, supernatant of *Sm*TPH in induction temperature of 18℃; 2, precipitate of *Sm*TPH in induction temperature of 18℃; 3, supernatant of *Sm*TPH in induction temperature of 25℃; 4, precipitate of *Sm*TPH in induction temperature of 25℃.


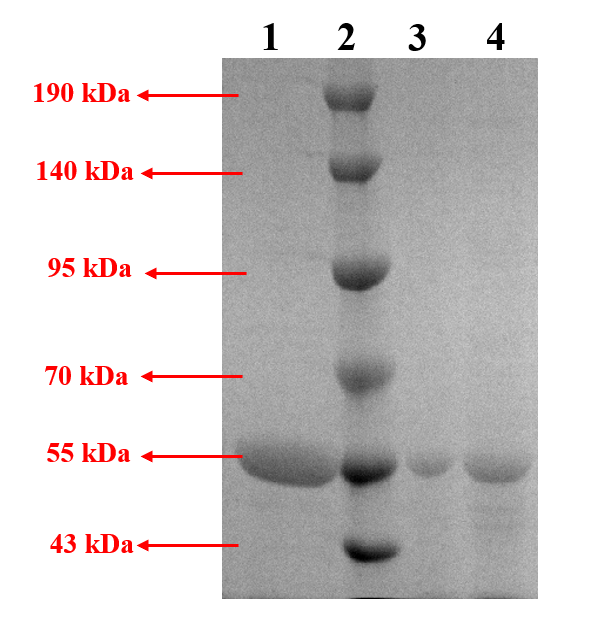


Fig. S2 SDS-PAGE of cell extracts of BL21(DE3)/pET24a-DDC. Line M is. Line 1, 2, 3, 4 are purified enzyme, protein size marker, precipitate and supernatant of sonicating cells cultured.





Fig. S3. Effects of different catalysis temperatures on the catalysis in 5-HTP of *Ha*DDC.





Fig. S4 Effects of different pH on the catalysis in 5-HTP of *Ha*DDC.





Fig. S5 Effects of different PLP concentration on the catalysis in 5-HTP of *Ha*DDC.


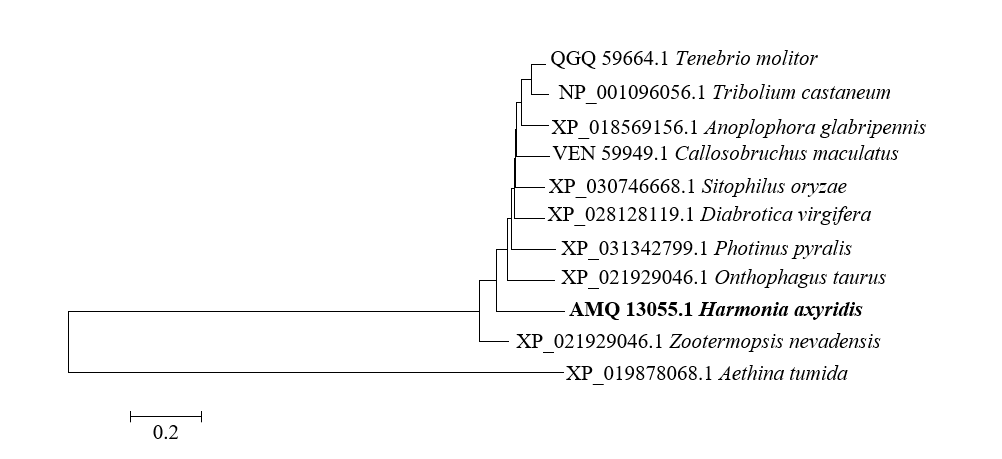


Fig.S6 Phylogenetic analysis of *Ha*DDC with other DDCs. It was highlighted with a black overstriking. The following proteins were used for analysis: *Tenebrio molitor* (QGQ59664.1), *Tribolium castaneum* (NP_001096056.1), *Diabrotica virgifera* (XP_028128119.1), *Sitophilus oryzae* (XP_030746668.1), *Anoplophora glabripennis* (XP_018569156.1), *Callosobruchus maculatus* (VEN59949.1), *Zootermopsis nevadensis* (XP_021929046.1), *Photinus pyralis* (XP_031342799.1), and *Onthophagus taurus* (XP_021929046.1).


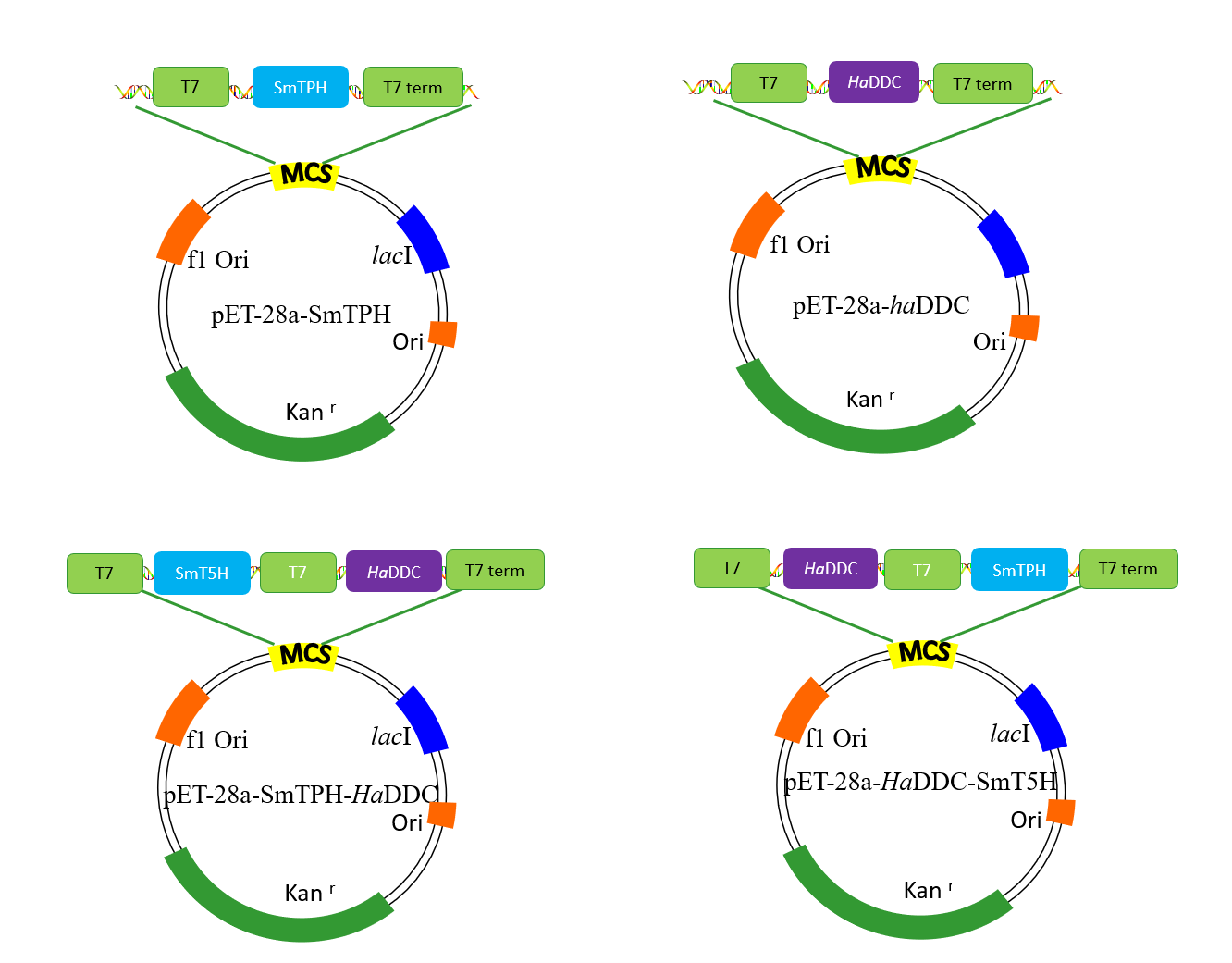


Fig.S7 Plasmids construction of *Sm*TPH gene and *Ha*DDC gene at different cloning sites in the pET-28a (+).

**Tab. S1** The synthesized protein sequence applied in this study.

| **Protein** | **Protein Sequence** |
| --- | --- |
| *Sm*TPH | MISTESDLRRQLDENVRSEADESTKEECPYINAVQSHHQNVQEMSIIISLVKNMNDMKSIISIFTDRNINILHIESRLGRLNMKKHTEKSEFEPLELLVHVEVPCIEVERLLEELKSFSSYRIVQNPLMNLPEAKNPTLDDKVPWFPRHISDLDKVSNSVLMYGKELDADHPGFKDKEYRKRRMMFADIALNYKWGQQIPIVEYTEIEKTTWGRIYRELTRLYKTSACHEFQKNLGLLQDKAGYNEFDLPQLQVVSDFLKARTGFCLRPVAGYLSARDFLSGLAFRVFYCTQYIRHQADPFYTPEPDCCHELLGHVPMLADPKFARFSQEIGLASLGTSDEEIKKLATCYFFTIEFGLCRQDNQLKAYGAGLLSSVAELQHALSDKAVIKPFIPMKVINEECLVTTFQNGYFETSSFEDATRQMREFVRTIKRPFDVHYNPYTQSIEIIKTPKSVAKLVQDLQFELTAINESLLKMNKEIRSQQFTTNKIVTENRSS |
| *Ha*DDC | MEANQFRDFGKAMIDYVANYLENIRERRVLPTVEPGYLRPLLPSEAPQKPDTWQEVMADIEKVIMPGVTHWHSPKFHAYFPTANSYPAIVADILSDGIACIGFSWIASPACTELEVVMMDWLGKMIGLPEEFLACSGGKGGGVIQGTASEATLVALLGAKARAIHHVKKEHPDWKDADIAEKLVGYTSSQSHSSVERAGLLGGVKLRGLPTDESNRLRGDTLERAIKEDREAGLIPFYVVATLGTTSSCTFDNLEEIGPVCNVNKVWLHIDAAYAGAAFTCPEYRYLMKGVEMADSFDFNPHKWMLVTFDCSAMWLKDPNWLVDAFNVDPLYLKHDQQGSAPDYRHWQIQLGRRFRALKIWFVLRLYGVENIQKHIRKQIGLAHHFEDLVKSDDRFEVTEEVLMGLVCFRLKGQSNEVNERLLKRINARGTIHLVPSKIREMYFLRMAVCSRLTEKEDMDLSWKEVRESADDILGE |
